# Chemoenzymatic synthesis of sialylated lactuloses and their inhibitory effects on *Staphylococcus aureus*

**DOI:** 10.1371/journal.pone.0199334

**Published:** 2018-06-20

**Authors:** Jie Zeng, Yajie Hu, Tian Jia, Ruiyao Zhang, Tongchao Su, Junliang Sun, Haiyan Gao, Guanglei Li, Meng Cao, Mengdi Song

**Affiliations:** School of Food Science, Henan Institute of Science and Technology, Xinxiang, China; National Cancer Institute at Frederick, UNITED STATES

## Abstract

**Background:**

Sialylated glycoconjugates play important roles in physiological and pathological processes. However, available sialylated oligosaccharides source is limited which is a barrier to study their biological roles. This work reports an efficient approach to produce sialic acid-modified lactuloses and investigates their inhibitory effects on *Staphylococcus aureus* (*S*. *aureus*).

**Methods:**

A one-pot two-enzyme (OPTE) sialylation system was used to efficiently synthesize sialylated lactuloses. Silica gel flash chromatography column was employed to purify the sialylated products. The purity and identity of the product structures were confirmed with mass spectrometry (MS) and nuclear magnetic resonance (NMR). The inhibitory effect of sialylated lactuloses against *S*. *aureus* was evaluated by using microplate assay, fluorescence microscopy, DAPI (4',6-diamidino-2-phenylindole) fluorescence staining and protein leakage quantification.

**Results:**

Neu5Ac-containing sialylated lactuloses with either α2,3- or α2,6-linkages were efficiently synthesized via an efficient OPTE sialylation system using α-2,3-sialyltransferase or α-2,6-sialyltransferase, respectively. Neu5Ac-α2,3-lactulose and Neu5Ac-α2,6-lactulose significantly inhibited the growth of *S*. *aureus*. Fluorescence microscopy and DAPI fluorescence staining indicated that the sialylated lactuloses might disrupt nucleic acid synthesis of *S*. *aureus*.

**Conclusions:**

Neu5Ac-containing sialylated lactuloses had higher antibacterial activity against *S*. *aureus* than non-sialylated lactulose. The inhibitory effect of Neu5Ac-α2,3-lactulose was superior to that of Neu5Ac-α2,6-lactulose. The sialylated lactuloses might inhibit *S*. *aureus* by causing cell membrane leakage and disrupting nucleic acid synthesis.

## Introduction

Lactulose [4-O-(β-D-galactopyranosyl)-D-fructose] is a non-natural disaccharide and is synthesized from lactose by isomerization under alkaline conditions [1] or using cellobiose 2-epimerase [2]. Lactulose can also be produced in the presence of fructose via β-galactosidase bioconversion [1, 3–4]. Possessing bifidogenic activity, lactulose has been used to treat constipation and some liver diseases in humans [5]. Lactulose is non-absorbable in the upper gastrointestinal tract but can be metabolized by colonic bacteria. Due to its prebiotic activity, lactulose is widely used in food industries as a functional ingredient [6].

Sialic acids are a family of α-keto acids with a nine-carbon backbone. They are usually present as terminal carbohydrate units of glycoproteins and glycolipids of vertebrates, components of capsular polysaccharides, and lipooligosaccharides of pathogenic bacteria [7]. Sialylated glycoconjugates play the important roles in physiological and pathological processes, including cellular recognition, bacterial infection, viral infection, and tumor cell metastasis, development and differentiation [8].

Today, the roles of intestinal microbiota for human health have been widely recognized. Sialylated oligosaccharides, which serve as receptors for pathogens, can inhibit the pathogen adherence to the intestinal epithelia [9–11]. Sialylated oligosaccharides can block the adhesion of *Helicobacter pylori*, which causes peptic ulcers and other gastric diseases, to intestinal cells [12]. The compound 3′-sialyllactose (Neu5Ac-α2,3Gal-β-1,4Glc)—a major constituent of both bovine milk oligosaccharides (BMOs) and human milk oligosaccharides (HMOs)—can inhibit the binding of *H*. *pylori* to human duodenal cells [12]. Isolated BMOs blocked the *in vitro* adhesion of seven enterotoxigenic *Escherichia coli* strains [13] and *Neisseria meningitidis* to the target cells [13–14]. BMOs are likely not as selective as HMOs because of their simpler structural decoration, though an increase in the degree of sialylation will improve their prebiotic selectivity.

A particular difficulty of the application of sialylated oligosaccharides is the lack of sufficient material for preclinical trials. Indeed, this scarcity of adequate sources of HMO-like molecules has been a major factor inhibiting their development as health products. Therefore, more production schema will be needed to produce novel synthetic sialylated oligosaccharides that are highly likely to possess antimicrobial action.

*Staphylococcus aureus (S*. *aureus)* produces a broad range of virulence factors that contribute to bacterial invasion and thus plays an important role among prevalent etiological agents in intramammary infection [15]. *S*. *aureus* is a highly virulent pathogen that can resist many antibacterial agents and causes various diseases in humans and animals, including skin abscesses, pneumonia, endocarditis, and osteomyelitis[16]. *S*. *aureus* is also resistant to many antibiotics, especially to methicillin and vancomycin [17–18]. As sialylated glycans are widely expressed on the cell surface, evaluation of their inhibitory effects of sialylated glycans against *S*. *aureus* will help in the identification of better ligands for the preventing infection by this bacterium.

In this study, we developed an efficient one-pot two-enzyme (OPTE) sialylation system to achieve the chemoenzymatic synthesis of Neu5Ac-containing sialylated lactuloses with α2,3- and α2,6-linkages from the commercially available monosaccharide Neu5Ac (as the donor substrate) and the disaccharide lactulose (as the acceptor substrate). The antibacterial activities of the new synthetic sialylated compounds against *S*. *aureus* were determined.

## Material and methods

### Materials and reagents

Lactulose was purchased from Carbosynth Ltd. (Berkshire, UK), CTP was obtained from the Hangzhou Meiya Pharmaceutical Co., Ltd (Hangzhou, China), and Neu5Ac was obtained from the Ningbo Hongxiang Bio-chem Co., Ltd (Ningbo, China). *Neisseria meningitidis* CMP-sialic acid synthetase (NmCSS), a *Pasteurella multocida* multifunctional α2-3-sialyltransferase 1 M144D mutant (PmST1 M144D), and α-2,6-sialyltransferase from a recombinant *Photobacterium damselae* (Pd26ST) [19,20] were kindly provided by the National Glycoengineering Research Center (NGRC) at Shandong University, Jinan, China. *S*. *aureus* was kindly provided by a biological lab at the School of Food Science, Henan Institute of Science and Technology, China.

Magnesium chloride (MgCl_2_), *p*-anisaldehyde, acetoxyacetyl chloride, ethyl acetate (EtOAc), methyl alcohol (MeOH), normal propyl alcohol (n-propanol), ammonia, ethyl alcohol (EtOH), acetic acid (HOAc), sodium chloride, glucose and sodium hydrogen sulfite were of analytical grade (AR). Lysogeny broth (LB) and agar powder were of biotech grade (BR) and purchased from the Beijing Aoboxing Bio-Tech Co., Ltd. (Beijing, China).

### Bacterial strains and culture conditions

*S*. *aureus* was routinely cultured in LB medium, LB liquid Medium was prepared as follows: LB powder (25 g) was dissolved in 1000 mL of distilled water, which was then sterilized at 121°C for 15 min. For LB solid Medium, 1.8 g of agar powder was added to liquid LB medium, which was then sterilized at 121°C for 15 min and cooled to room temperature.

*S*. *aureus* was aerobically grown at 37°C in liquid LB medium with shaking at 190 revolutions per minute (rpm).

### Synthesis of Neu5Ac-α2,3-lactulose and Neu5Ac-α2,6-lactulose

Neu5Ac-α2,3-lactulose and Neu5Ac-α2,6-lactulose were synthesized from lactulose as the acceptor substrate and Neu5Ac as the donor precursor using a one-pot two-enzyme system containing NmCSS and a sialyltransferase (PmST1 for producing Neu5Ac-α2,3-lactulose and Pd26ST for producing Neu5Ac-α2,6-lactulose. (**S1 Fig**).

### Small assay OPTE sialylation reactions

As described in detail previously [21], the assays were performed in a total volume of 10 μL in Tris-HCl buffer (100 mM) with pH values varying from 7.0 to 9.0; the reaction contained lactulose (10 mM), Neu5Ac (15 mM), CTP (15 mM), MgCl_2_ (20 mM), NmCSS (5 μg), and PmST1_M144D or Pd26ST (5 μg). The reactions were allowed to proceed at 32, 37 and 42°C and monitored by thin-layer chromatography (TLC) every 30 min. All the assays were carried out in duplicate.

### Preparative-scale OPTE sialylation reactions

Lactulose (100 mg), CTP (246.7 mg), and Neu5Ac (135.4 mg) were placed in 10 mL of 100 mM Tris-HCl (pH 8.5) with 20 mM MgCl_2_, and NmCSS (4 mg), and PmST1_M144D (4 mg) (for synthesis of Neu5Ac-α2,3-lactulose) or Pd26ST (4 mg) (for synthesis of Neu5Ac-α2,6-lactulose) was added. The reaction was performed at 37°C overnight in a THZ-A air bath thermostat oscillator (Haixing Kedian Technology Co. Ltd. Shenzhen, China). The reactions were monitored by TLC (TLC developing solvent: EtOAc:MeOH:H_2_O:HOAc = 5:2:1:0.2 or n-Propanol:H_2_O:NH_3_·H_2_O = 5:2:1, by volume). When the reaction was completed, the same volume of 95% EtOH was added to the reaction mixture, which was then maintained at 4°C for 30 min and subsequently centrifuged (7000 rpm, 30 min) using an Avanti J-25 refrigerated centrifuge (Beckman Coulter, Inc., America). The supernatant was concentrated using a ZT-52C rotary evaporator (Zituo Instrument Equipment Co., LTD, Zhengzhou, China) and purified by silica gel flash column chromatography.

### Purification of sialylated lactuloses by silica gel flash column chromatography

EtOAc:MeOH:H_2_O (6:2:1, then 5:2:1) was employed as the silica gel eluting solvent. The eluted liquid was checked by TLC, and EtOAc:MeOH:H_2_O:HOAc (6:2:1:0.2) was used as the developing solvent. The fractions containing pure products were collected, concentrated using a rotary evaporator, and then dried by an Alpha 1-4LSC vacuum freeze drying instrument (Marin Christ, Osterode, Germany).

### Mass spectrum characterization of sialylated lactuloses

The purified sample (0.5 mg) was dissolved in 100 μL of distilled water at room temperature. Mass spectrum analyses were carried out on a tandem quadrupole detector (TQD) liquid chromatography mass spectrometry (LC/MS) system (Waters, USA) equipped with a vacuum degasser, quaternary pump, autoinjector, column compartment, diode-array detector (DAD), and ion-trap mass spectrometer with an electrospray ionization (ESI) interface. Scan ranges were from 150−800 m/z.

### NMR characterization of sialylated lactuloses

Purified sample (15 mg) was dissolved in 0.5 mL of D_2_O at room temperature. NMR spectra was recorded on a Bruker Avance-800 NMR spectrometer (800 MHz for ^1^H, 200 MHz for ^13^C).

### Antibacterial assays of synthesized α2,3- and α2,6-sialyllactuloses

Microplate assays were used for measuring the growth inhibition of *S*. *aureus* in accordance with He et al. [22] with some modifications. First, 100 μL of an *S*. *aureus* cell suspension (overnight cultures diluted to OD = 0.05) was added into liquid medium in wells of a 96-well microplate (Costar 3599, Corning Incorporated, USA) with different concentrations (8 mg/mL) of α2,3- and α2,6-sialyllactulose in the wells of a 96-well microplate (Costar 3599, Corning Incorporated, USA). The microplates were then placed in a biochemical incubator for 24 h at 37°C. Microplates were gently shaken in the incubator except during measurements of the OD value at 600 nm made every two hours by a Varioskan Flash instrument (Thermo Fisher, New York, USA). Control experiments (ck), 8 mg/mL lactulose and 8 μg/mL antibiotic kanamycin were conducted at the same time without sialylated lactuloses.

### Fluorescence microscopy

Single colonies of *S*. *aureus* were added to LB liquid medium and shock cultured for 8 h; a fraction of this culture was then added to LB liquid medium and incubated until the OD_600_ was 0.05. Next, 90 μL of this *S*. *aureus* cell suspension was placed in a 96-well microplate, and 10 μL of 8 mg/mL Neu5Ac-α2,3-lactulose or Neu5Ac-α2,3-lactulose was added to the wells. The microplate was incubated in a thermoshaker (MB100, Hangzhou, China) at 37°C for 8 h at 500 rpm. The same volume of 5 μg/mL 4,6-diamidino-2-phenylindole (DAPI, Invitrogen, Carlsbad, CA) was then added, and the samples was mixed uniformly and kept for 10 min in the dark. Fluorescence was observed using an inverted fluorescence microscope with a Zeiss-Series 120Q light source (America), and the sample was visually inspected for color changes. A 100-μL sample of OD_600_ 0.05 *S*. *aureus* without sialyllactulose was used as a control (ck).

### DNA content of *S*. *aureus*

*S*. *aureus* cell suspensions treated with different sialyllactuloses in the wells of the 96-well microplate were incubated at 37°C for 8 h. Next, 100 μL of the *S*. *aureus* cell suspension was collected, and the OD_600_ was adjusted to approximately 0.6. DAPI (5 μg/mL) was added three times in a row, and the samples were allowed to stand in the dark for 15 min. After two washes with phosphate-buffered saline (PBS), the fluorescence of the samples was measured using a Fluorescence spectrophotometer (Cary Eclipse G9800A, Agilent Technologies, America) at an emission wavelength of 454 nm and excitation at 364 nm. *S*. *aureus* without sialyllactulose was used as the control.

### Protein leakage in cell membranes

*S*. *aureus* cell suspensions treated with different sialyllactuloses were incubated at 37°C for 8 h and centrifuged for 5 min at 8000 rpm using a Thermo Scientific refrigerated centrifuge (Multifuge X1R, Heraeus, Germany). A 0.1 mL aliquot of supernatant was transferred by pipette to a test tube, and 1 mL of G-250 Coomassie brilliant blue solution was added. The solution was blended and allowed to stand for 5 min after which 0.1 mL of mixture was absorbed to 96-well plates. Protein leakage was determined by measuring the optical density of the cell supernatants at 595 nm using a microplate reader (Infinite F50, Tecan, Switzerland). A standard curve (y = 17.106x + 0.2698. y, absorbance; x, content of protein) was used to calculate the amount of protein leakage content.

### Statistical analysis

Data obtained from the experiments were analyzed using Microsoft Excel 2007 to determine statistical significances. Differences were considered statistically significant at P < 0.05.

## Results

### Small reaction assays

**Fig 1:**
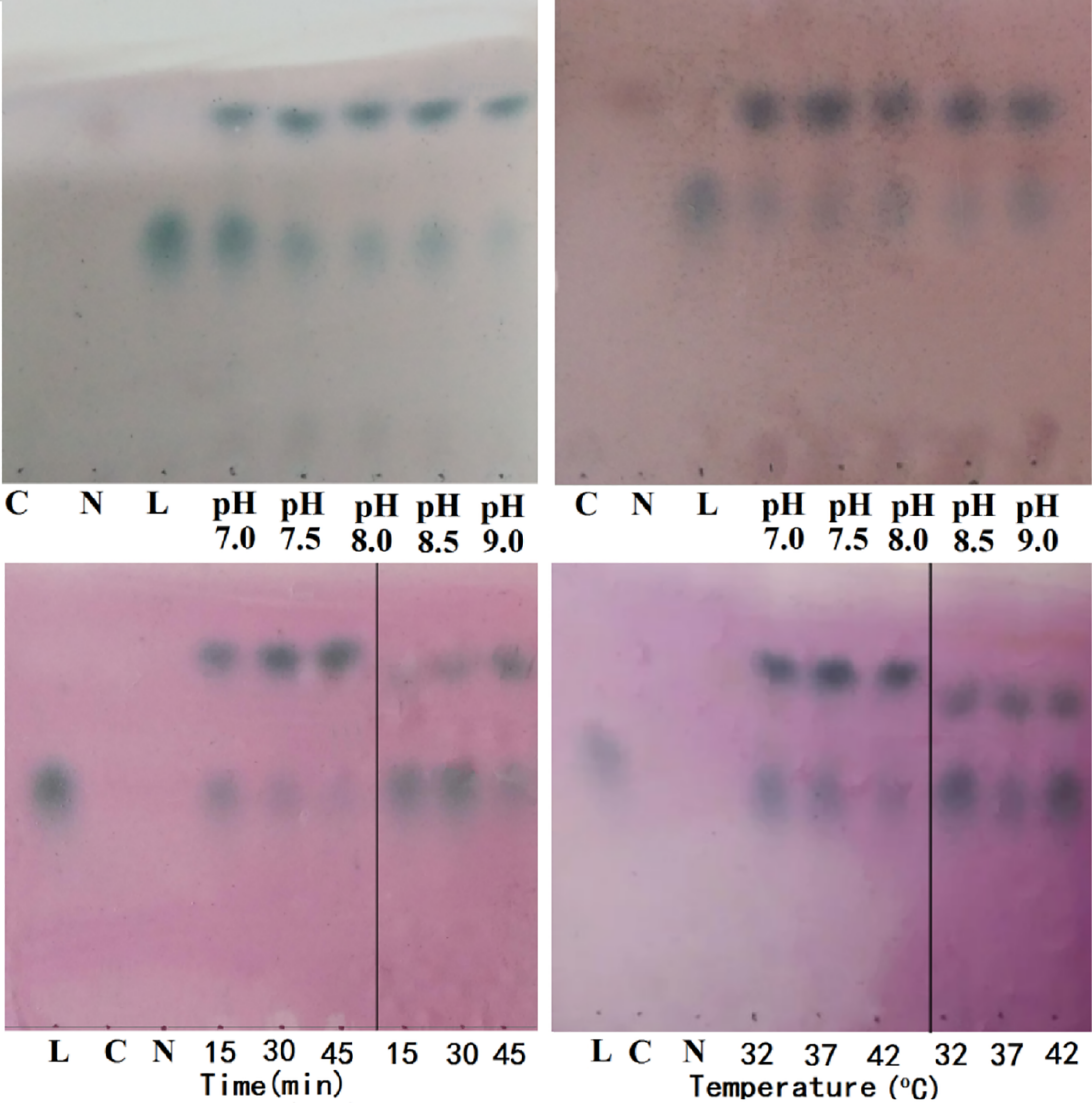
Synthesis conditions of sialyllactuloses by TLC analysis. L, C, N in every figure were standards. L, lactulose; C, CTP; N, Neu5Ac; (a), synthesis of Neu5Ac-α2,3-lactulose at different pH; (b), synthesis of Neu5Ac-α2,6-lactulose at different pH; (c), synthesis of Neu5Ac-α2,3-lactulose and Neu5Ac-α2,6-lactulose for different time; (d), synthesis of Neu5Ac-α2,3-lactulose and Neu5Ac-α2,6-lactulose at different temperatures. In Fig 1C and 1D, in the vertical bar on the left is reaction of Neu5Ac-α2,3-lactulose, on the right is reaction of Neu5Ac-α2,6-lactulose.

As shown in **Fig 1**, lactulose was used as a sialyltransferase acceptor, and α2,3-sialylated and α2,6-sialylated trisaccharides were readily produced from lactulose by carrying out an efficient two-enzyme sialylation approach in sequence. As presented in **Fig 1A** and **Fig 1B,** the yields showed that the sialylation yields at 37°C for 45 min at pH 8.0 and 9.0 were higher than those at pH 7.0 and 7.5. **Fig 1C** showed that the efficiency of producing Neu5Ac-α2,3-lactulose was higher than that of producing Neu5Ac-α2,6-lactulose at 37°C and pH 8.5, which can be attributed to the higher activity of the enzyme PmST1_M144D. According to the data depicted in **Fig 1D,** the activity of PmST1 was slightly higher at 42°C than at 37°C, though the activity of Pd26ST was higher at 37°C than at 42°C. These results were coincided with the results of a previous report (Yu et al, 2005).

### Mass spectra

**Fig 2:**
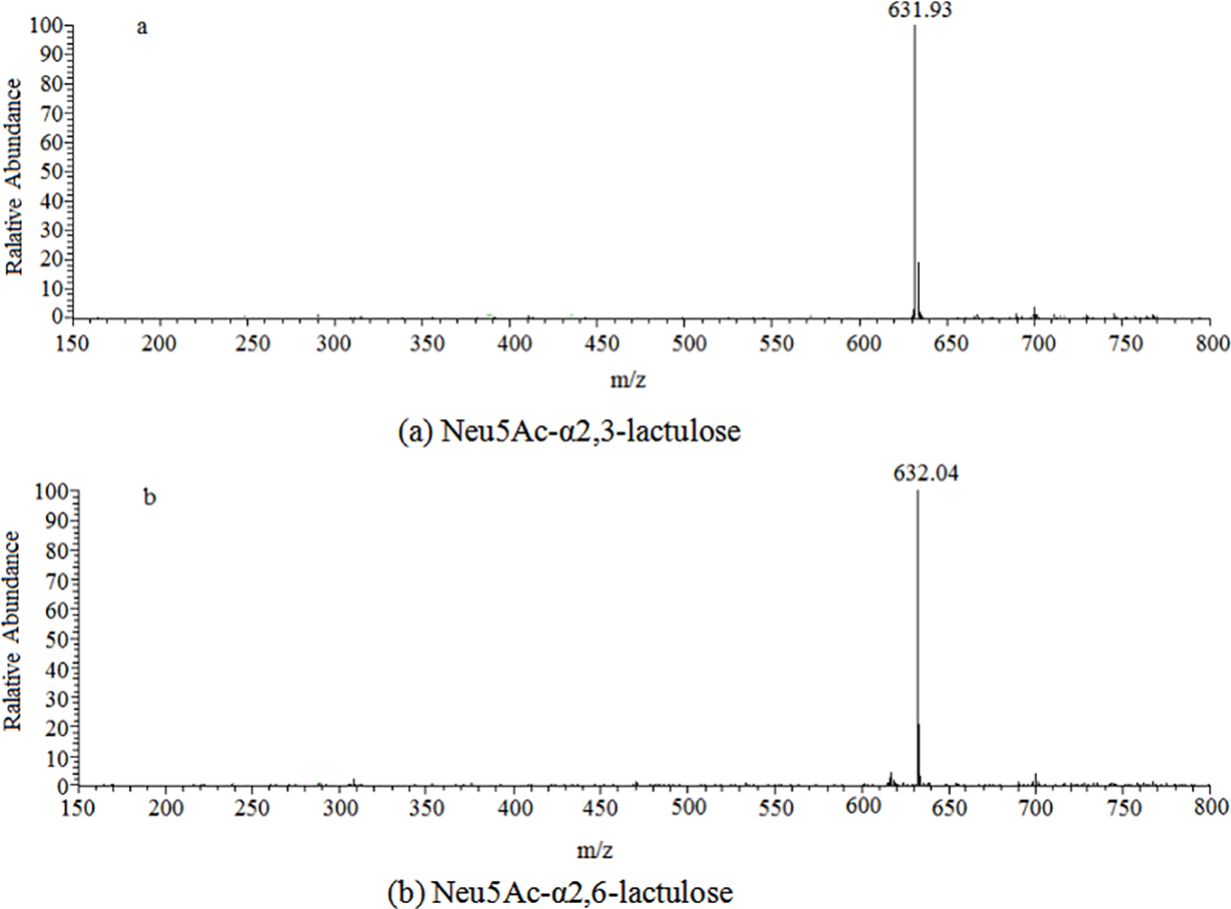
Mass spectrum of purified Neu5Ac-α2,3-lactulose and Neu5Ac-α2,6-lactulose.

Large-scale reactions were conducted based on the results of the small reactions. The conditions of the synthesis reactions were 42°C and pH 8.5 for PmST1_M144D-catalyzed reactions, and at 37°C and pH 8.5 for Pd26ST-catalyzed reactions. The sialylated products were purified by silica gel flash chromatography. The MS spectra of the pure products are illustrated in **Fig 2**. The molecular weights of the oligosaccharides were found to be 632 Da (**Fig 2A and 2B**, lactulose 342 Da). The mass results indicated that sialylated lactuloses were obtained from both PmST1_M144D- and Pd26ST-catalyzed enzymatic reactions.

### NMR characterization

The ^1^H NMR spectrum of Neu5Ac-α2,3-lactulose displayed three β-anomeric galactosyl protons at δ 4.65, 4.55 and 4.53 in a ratio of 68%:23%:9%, indicating an equilibrium mixture of Neu5Ac-α2,3-lactulose isomers, Neu5Ac-α2,3-Gal-β1,4-β-D-fructopyranose (a), Neu5Ac-α2,3-Gal-β1,4-β-D- fructofuranose (b), and Neu5Ac-α2,3-Gal-β1,4-α-D-fructofuranose (c) (**S2 Fig**). Similarly, Neu5Ac-α2,6-lactulose exhibits three β-anomeric galactosyl protons at δ 4.54, 4.46 and 4.43 in a ratio of 60%:28%:12%, indicating an equilibrium mixture of Neu5Ac-α2,6-lactulose isomers, Neu5Ac-α2,6-Gal-β1,4-β-D-fructopyranose (a’), Neu5Ac-α2,6-Gal-β1,4-β-D-fructofuranose (b’), Neu5Ac-α2,3-Gal-β1,4-α-D-fructofuranose (c’) (**S3 Fig**). These results were consistent with previous NMR study of lactulose, which mainly consists of three lactulose isomers: 4-*O*-(β-D-galactopyranosyl)-β-D-fructopyranose, 4-*O*-(β-D-galactopyranosyl)-β-D-fructofuranose, 4-*O*-(β-D-galactopyranosyl)-α-D-fructofuranose, with ration 66%: 25%: 9% in aqueous solution [23].

### Antibacterial assays with α2,3- and α2,6-sialyllactuloses

**Fig 3:**
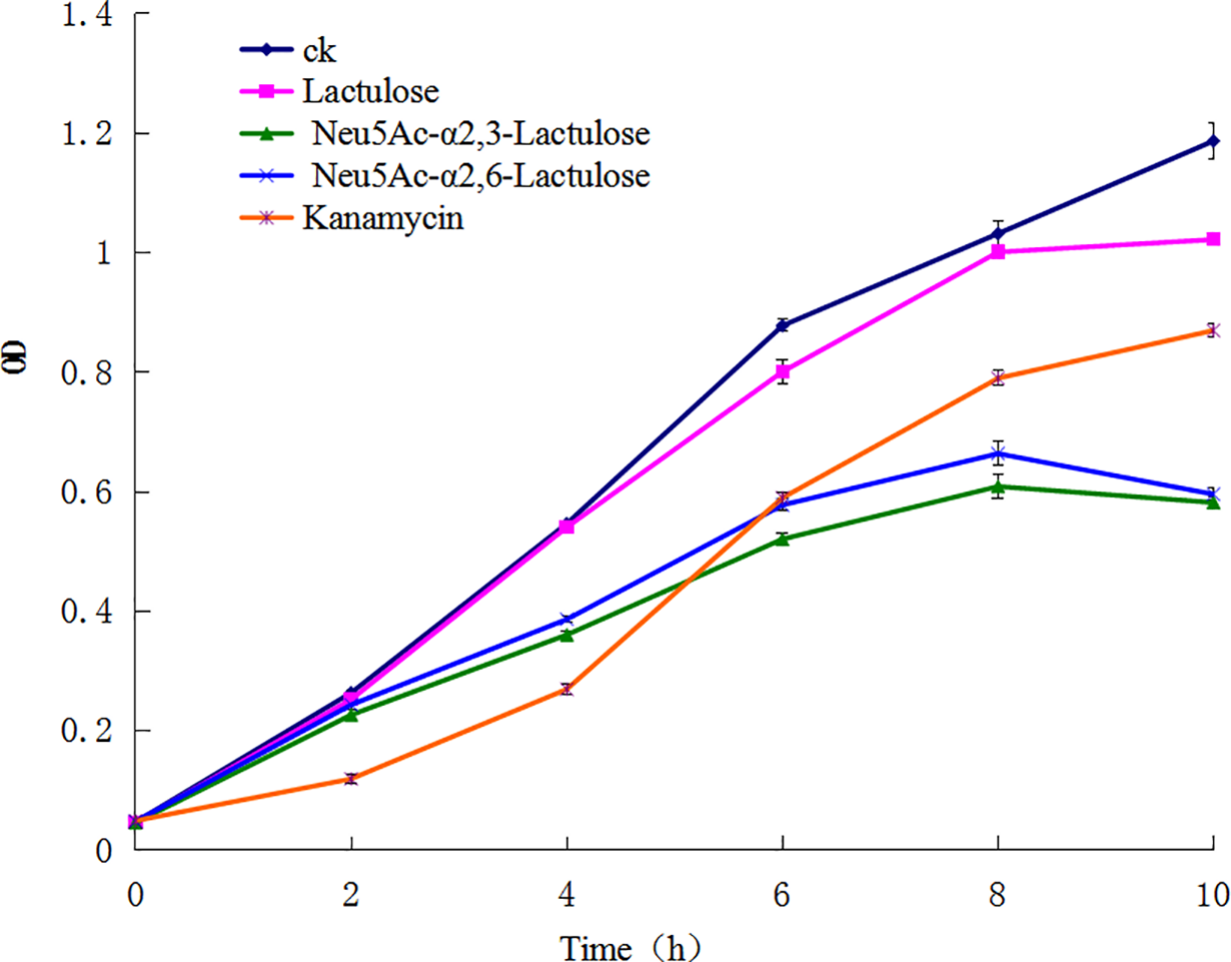
The bacterial growing curves of *S*. *aureus* in modified medium containing α2,3-linked and α2,6-linked sialyllactuloses. The control strain was not added sialyllactulose. The results are the mean values obtained from three separate experiments. Error bars represent the standard deviation.

Growth of different cultures on modified liquid medium supplemented with 8 mg/mL sialyllactulose with either α2,3- or α2,6- linkages was assessed by measuring the OD_600_ for 12 hours under aerobic conditions **(Fig 3)**. Neu5Ac-α2,3-lactulose and Neu5Ac-α2,6-lactulose clearly inhibited the growth of *S*. *aureus*, and the curves indicated the typical characteristics of concentration-dependent antibiotic activity. The growth of *S*. *aureus* treated with sialyllactulose was slightly slower than that of the control and lactulose groups after 2 h, and a death phase began at 10 h **(Fig 3)**. In addition, *S*. *aureus* was greatly inhibited by antibiotic kanamycin for 6 h, though the inhibitory effects of the two sialyllactuloses surpassed those of the kanamycin after 6 h. That is, inhibition of *S*. *aureus* by 8 mg/mL sialyllactulose with either α2,3- or α2,6- linkages may be better than that of 8 μg/mL kanamycin. Therefore, sialyllactuloses may serve as functional oligosaccharides beneficial to the intestinal tract.

### Fluorescence microscopy

**Fig 4:**
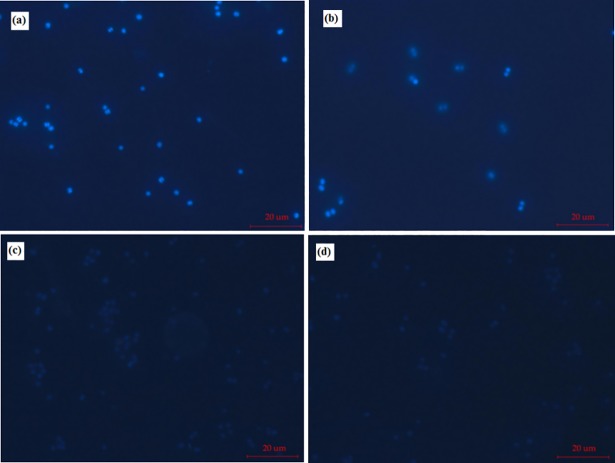
Changes of fluorescence intensity of *S*. *aureus* strains. a, b, c and d were the samples with different carbohydrates. a, control; b, lactulose; c, α2,3-linked sialyllactulose; d, α2,6-linked sialyllactulose.

DAPI is a fluorescent dye that binds to DNA of *S*. *aureus* strains and used to indicate cell viability, because it can penetrate the cell to enter living cells. Larger amounts of nucleic acids result in stronger blue fluorescence, which indicates that cells are viable, i.e., a visible change from blue fluorescence emittance to a lack of emittance indicates cell growth inhibition. **Fig 4** shows that the fluorescence intensity of experimental groups treated with α2,3- and α2,6-linked sialyllactuloses was weaker than that of the control and lactulose groups. The fluorescent intensity of the lactulose group was slightly weaker than that of the control.

### DNA content of *S*. *aureus*

**Fig 5:**
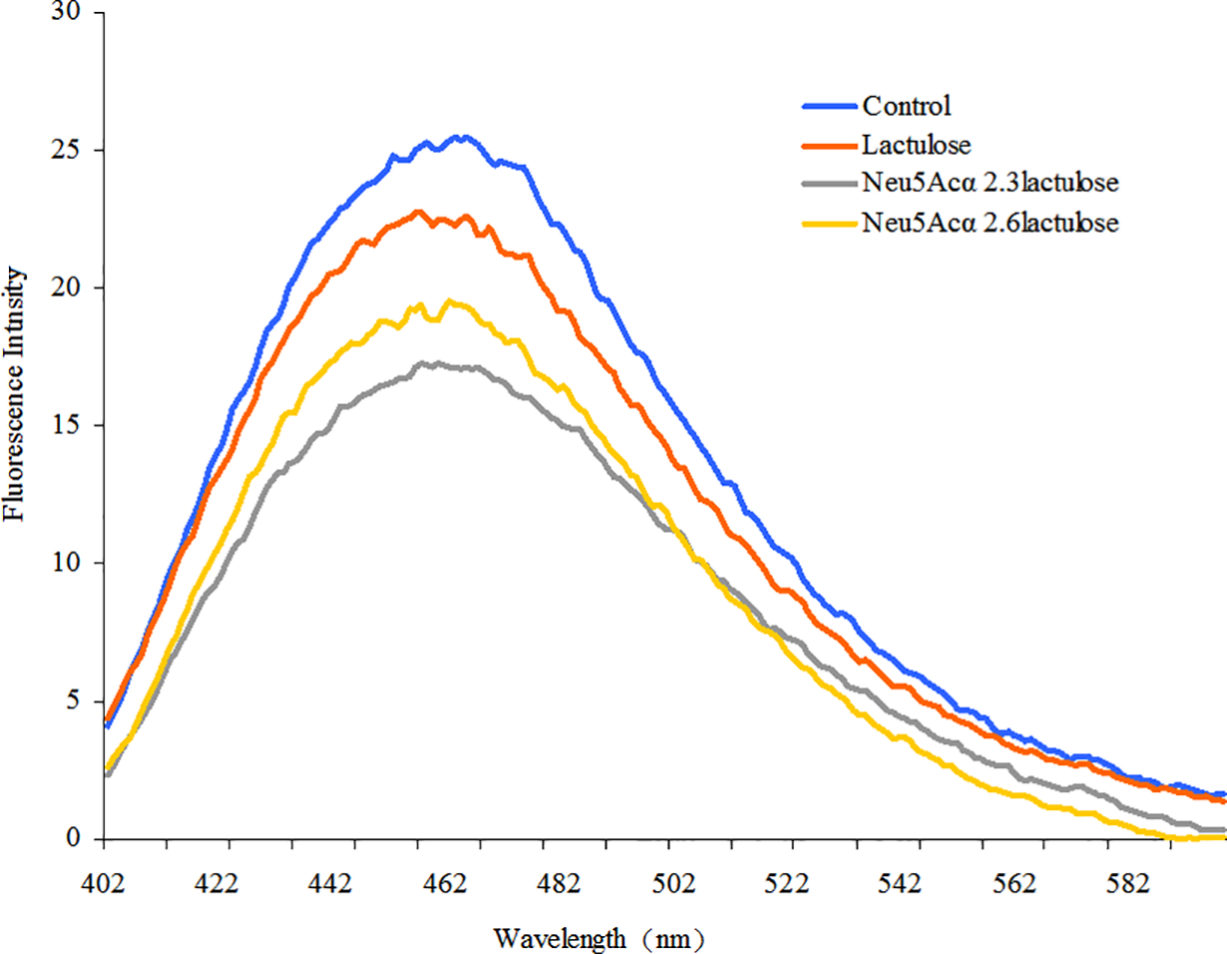
Changes of DNA content of *S*. *aureus with* α2,3-linked and α2,6-linked sialyllactuloses.

Changes in the DNA content of *S*. *aureus* with or without sialyllactuloses were determined by measuring the fluorescece intensity between 400 and 600 nm, and the spectra were presented in **Fig 5**. The maximum excitation and emission wavelengths of DAPI fluorescence bound to double-stranded DNA were 364 and 454 nm, respectively. As shown in **Fig 5,** the DNA content of *S*. *aureus* treated with lactulose and α2,3- and α2,6-linked sialyllactuloses was less than that of the control groups. The fluorescence intensities was 11.6, 32.7 and 23.8% lower than that of the control group, respectively. These results indicated that sialyllactulose interfered with nucleic acid synthesis in *S*. *aureus*.

### Protein leakage

**Fig 6:**
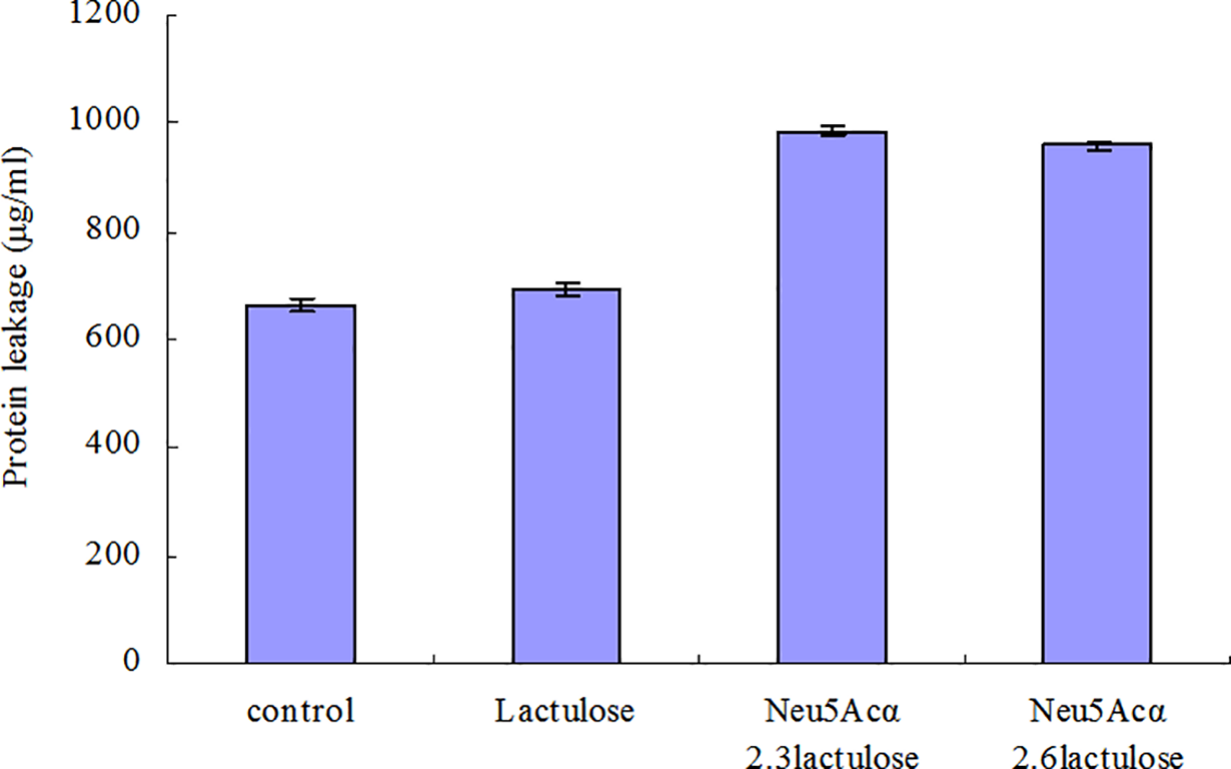
Protein leakage in *S*. *aureus* cells exposed to α2,3-linked and α2,6-linked sialyllactuloses.

The effects of sialyllactulose on *S*. *aureus* cell structures and membranes were used to investigate the antimicrobial mode of sialyllactulose. To this end, protein leakage induced by cell membrane damage was assessed (**Fig 6**). The protein leakage from *S*. *aureus* exposed to α2,3-and α2,6-linked sialyllactuloses (986.2 and 958.6 μg/mL, respectively) was higher than that from samples exposed to lactulose and control samples (664.73 and 693.2 μg/mL, respectively). The result suggested that the exposure of *S*. *aureus* cells to sialyllactulose induced membrane damage, resulting in the leakage of intracellular proteins.

## Discussions

HMOs have various antimicrobial activities. Some of them are receptors for pathogens because the HMOs share structural motifs with glycans on the infant intestinal epithelia. HMOs might therefore act as competitive inhibitors for the normal intestinal epithelial cell membrane binding sites to prevent binding by pathogenic bacteria [9]. The competitive inhibition of HMOs for adhesion of pathogens to intestinal cell membrane glycoconjugates [24, 25] may limit the virility of the latter in immuno-compromised infants, and may be responsible for the lower rates of diarrheal infection being lower in breast-fed infants compared to formula-fed infants [26]. When incubated with intestinal cells *in vitro*, sialylated oligosaccharides reduced the binding of a variety of pathogenic bacteria to target cells [10, 11]. Nonetheless, currently available synthetic HMO sources are limited and expensive, and also fail to represent the natural breadth of the large and complex structures present in human milk.

In this study, two HMO-mimics, Neu5Ac-containing sialylated lactuloses with either an α2,3- or α2,6-linkage, were efficiently synthesized via an efficient OPTE sialylation system. They both showed better antibacterial activity against *S*. *aureus* than non-sialylated lactulose. Although these new sialylated lactuloses exhibited unremarkable antibacterial activities, our work greatly advances HMO-mimic design. In addition, we plan to explore immunocompetence properties and to synthesize a greater number of HMO-mimics on the basis of the these two Neu5Ac-containing sialylated lactuloses with the aim of increasing their antibacterial activities.

## Conclusions

In conclusion, novel sialyllactuloses with α2,3-linkage (Neu5Ac-α2,3-lactulose) and α2,6-linkage (Neu5Ac-α2,6-lactulose) were efficiently synthesized from Neu5Ac and lactulose using an OPTE sialylation approach. These two novel sialyllactuloses showed some antimicrobial activity against *S*. *aureus*. Fluorescence microscopy and the changes in DNA content indicated that these sialyllactuloses interfered in the synthesis of DNA during the growth of *S*. *aureus*. The amount of protein leakage suggested that the antibacterial mechanism of these two sialyllactuloses involves the disruption of membrane integrity in *S*. *aureus* cells. The results of inhibition assays showed that these sialylated lactuloses had better antibacterial activity against *S*. *aureus* than lactulose.

## Supporting information

S1 Fig Enzymatic synthesis of Neu5Ac-α2,3-lactulose and Neu5Ac-α2,6-lactulose via a one-pot two-enzyme sialylation system. (PDF)

S2 Fig ^1^H, ^13^C, and COSY NMR spectra of Neu5Ac-α2,3-lactulose. A, ^1^H NMR spectra; B, zoomed ^1^H NMR spectra; a: Neu5Ac-α2,3-Gal-β1,4-β-D-fructopyranose; b: Neu5Ac-α2,3-Gal-β1,4-β-D-fructopyranose; c: Neu5Ac-α2,3-Gal-β1,4-α-D-fructopyranose, with ration 68%: 23%: 9%; C, ^13^C NMR spectra.
(PDF)

S3 Fig ^1^H, ^13^C, and COSY NMR spectra of Neu5Ac-α2,6-lactulose. A, ^1^H NMR spectra; B, zoomed ^1^H NMR spectra; a’: Neu5Ac-α2,6-Gal-β1,4-β-D-fructopyranose; b’: Neu5Ac-α2,6-Gal-β1,4-β-D-fructopyranose; c’: Neu5Ac-α2,6-Gal-β1,4-α-D-fructopyranose, with ration 60%: 28%: 12%; C, ^13^C NMR spectra.
(PDF)

S1 Data 1) NMR data of Neu5Ac-α2,3-lactulose; 2) NMR data of Neu5Ac-α2,6-lactulose. (PDF)
