# Correction: Chemoenzymatic synthesis of sialylated lactuloses and their inhibitory effects on *Staphylococcus aureus*

**DOI:** 10.1371/journal.pone.0204466

**Published:** 2018-09-17

**Authors:** The PLOS ONE Staff

In the Funding section, the grant number from the funder Excellent Youth Foundation of He’nan Scientific Committee is listed incorrectly. The correct grant number is: 174100510003. The publisher apologizes for the error.
