# Supplementary material for: Chemoenzymatic synthesis of sialylated lactuloses and their inhibitory effects on Staphylococcus aureus
Source: PLoS One. 2018 Jun 20;13(6):e0199334. doi: 10.1371/journal.pone.0199334 (PMC6010273; doi:10.1371/journal.pone.0199334)
Supplement: S1 Fig — (PDF) [file pone.0199334.s001.pdf]

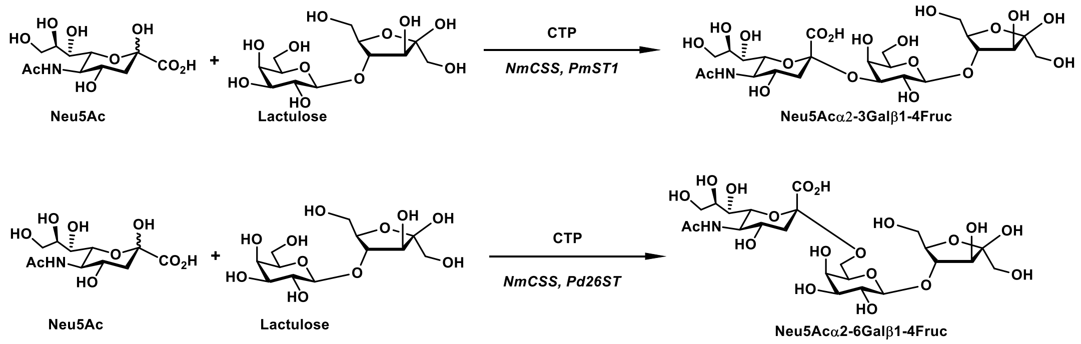

S1 Fig. Enzymatic synthesis of Neu5Ac-α2,3-lactulose and Neu5Ac-α2,6-lactulose via a one-pot two-enzyme sialylation system.
