# Supplementary material for: Chemoenzymatic synthesis of sialylated lactuloses and their inhibitory effects on Staphylococcus aureus
Source: PLoS One. 2018 Jun 20;13(6):e0199334. doi: 10.1371/journal.pone.0199334 (PMC6010273; doi:10.1371/journal.pone.0199334)
Supplement: S2 Fig — A, 1H NMR spectra; B, zoomed 1H NMR spectra; a: Neu5Ac-α2,3-Gal-β1,4-β-D-fructopyranose; b: Neu5Ac-α2,3-Gal-β1,4-β-D-fructopyranose; c: Neu5Ac-α2,3-Gal-β1,4-α-D-fructopyranose, with ration 68%: 23%: 9%; C, 13C NMR spectra. (PDF) [file pone.0199334.s002.pdf]

A

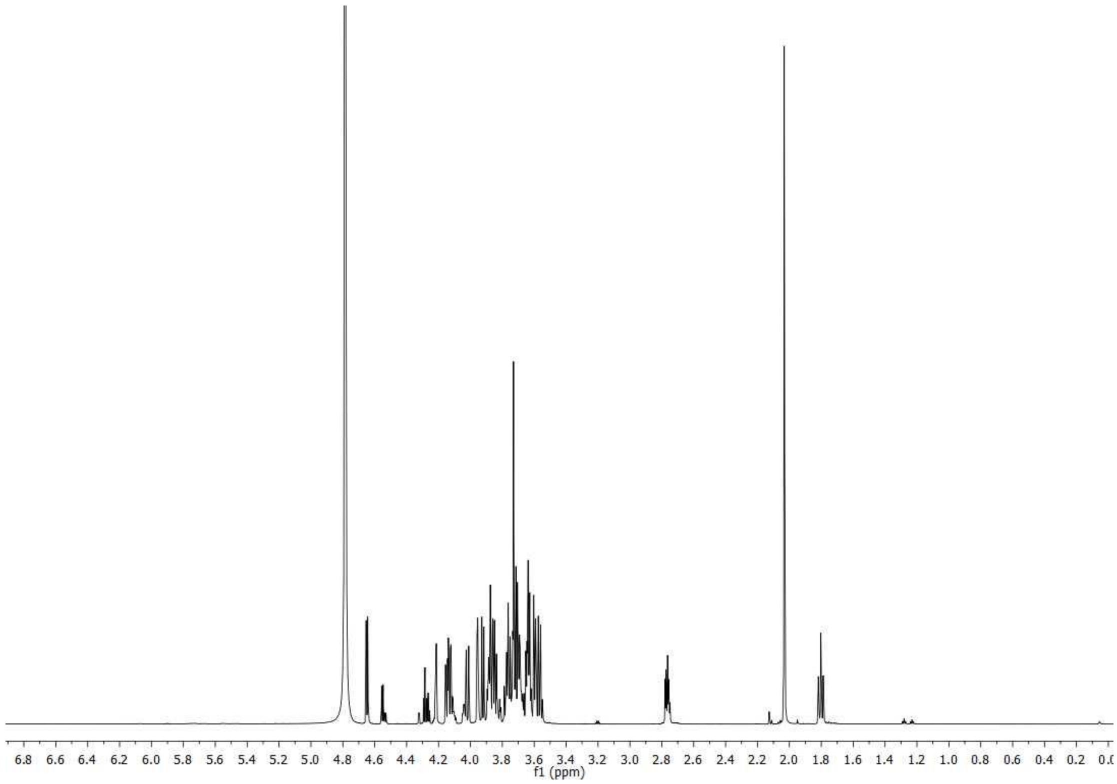
6.8 6.6 6.4 6.2 6.0 5.8 5.6 5.4 5.2 5.0 4.8 4.6 4.4 4.2 4.0 3.8 3.6 3.4 3.2 3.0 2.8 2.6 2.4 2.2 2.0 1.8 1.6 1.4 1.2 1.0 0.8 0.6 0.4 0.2
f1 (ppm)

B

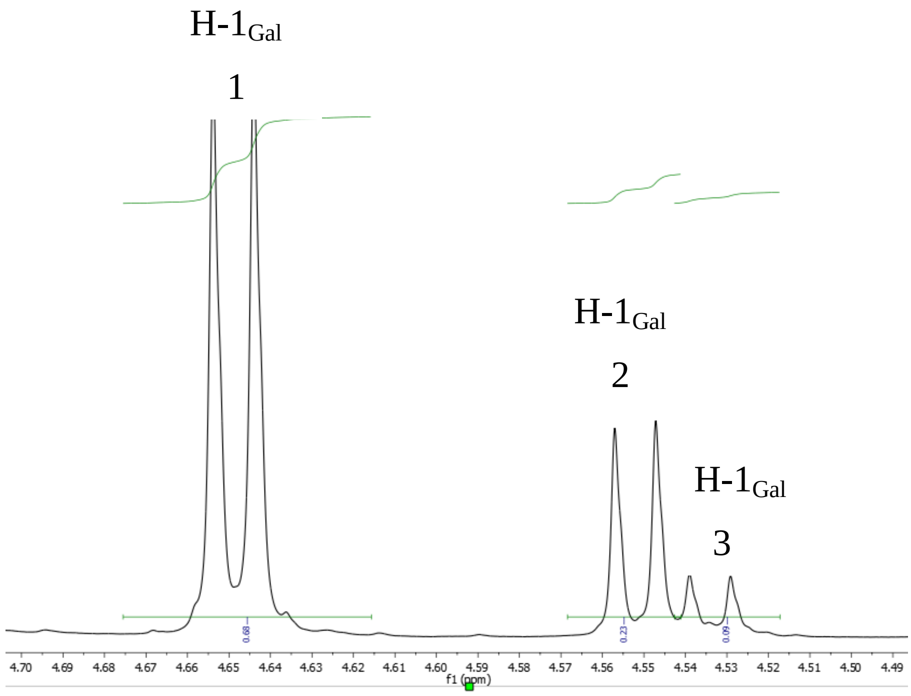
H-1Gal
1
H-1Gal
2
H-1Gal
3
0.68
0.23
0.09
4.70 4.69 4.68 4.67 4.66 4.65 4.64 4.63 4.62 4.61 4.60 4.59 4.58 4.57 4.56 4.55 4.54 4.53 4.52 4.51 4.50 4.49
f1 (ppm)

C

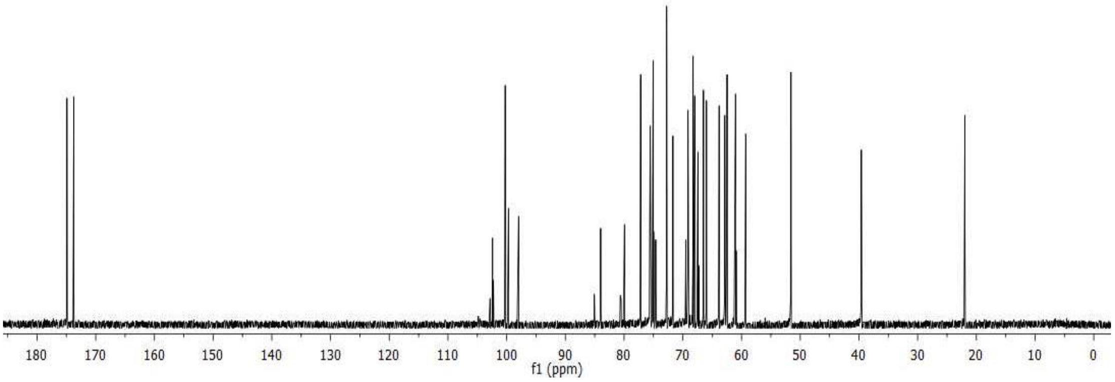

D

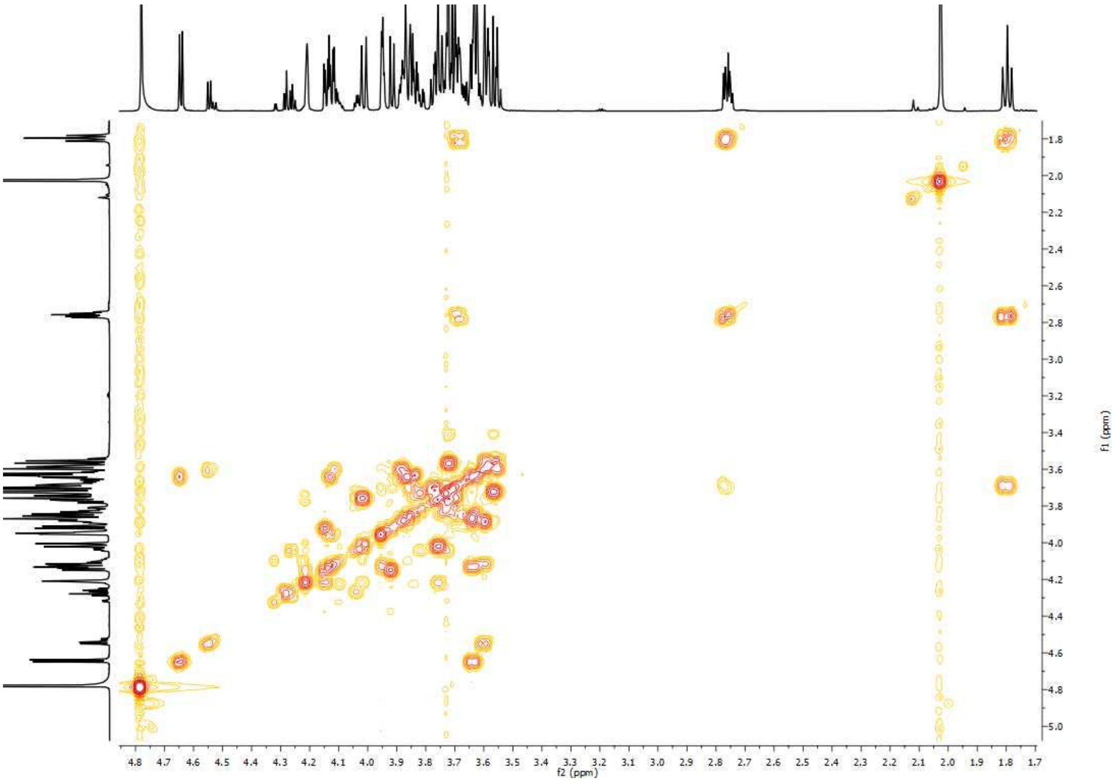

S2 Fig. ^{1}H, ^{13}C, and COSY NMR spectra of Neu5Ac-α2,3-lactulose. A, ^{1}H NMR spectra; B, zoomed ^{1}H NMR spectra; isomer 1: Neu5Ac-α2,3-Gal-β1,4-β-D-fructopyranose; isomer 2: Neu5Ac-α2,3-Gal-β1,4-β-D-fructopyranose; isomer 3: Neu5Ac-α2,3-Gal-β1,4-α-D-fructopyranose, with ration 68%: 23%: 9%; C, ^{13}C NMR spectra; D, COSY NMR spectra.
