# Supplementary material for: Chemoenzymatic synthesis of sialylated lactuloses and their inhibitory effects on Staphylococcus aureus
Source: PLoS One. 2018 Jun 20;13(6):e0199334. doi: 10.1371/journal.pone.0199334 (PMC6010273; doi:10.1371/journal.pone.0199334)
Supplement: S3 Fig — A, 1H NMR spectra; B, zoomed 1H NMR spectra; a’: Neu5Ac-α2,6-Gal-β1,4-β-D-fructopyranose; b’: Neu5Ac-α2,6-Gal-β1,4-β-D-fructopyranose; c’: Neu5Ac-α2,6-Gal-β1,4-α-D-fructopyranose, with ration 60%: 28%: 12%; C, 13C NMR spectra. (PDF) [file pone.0199334.s003.pdf]

A

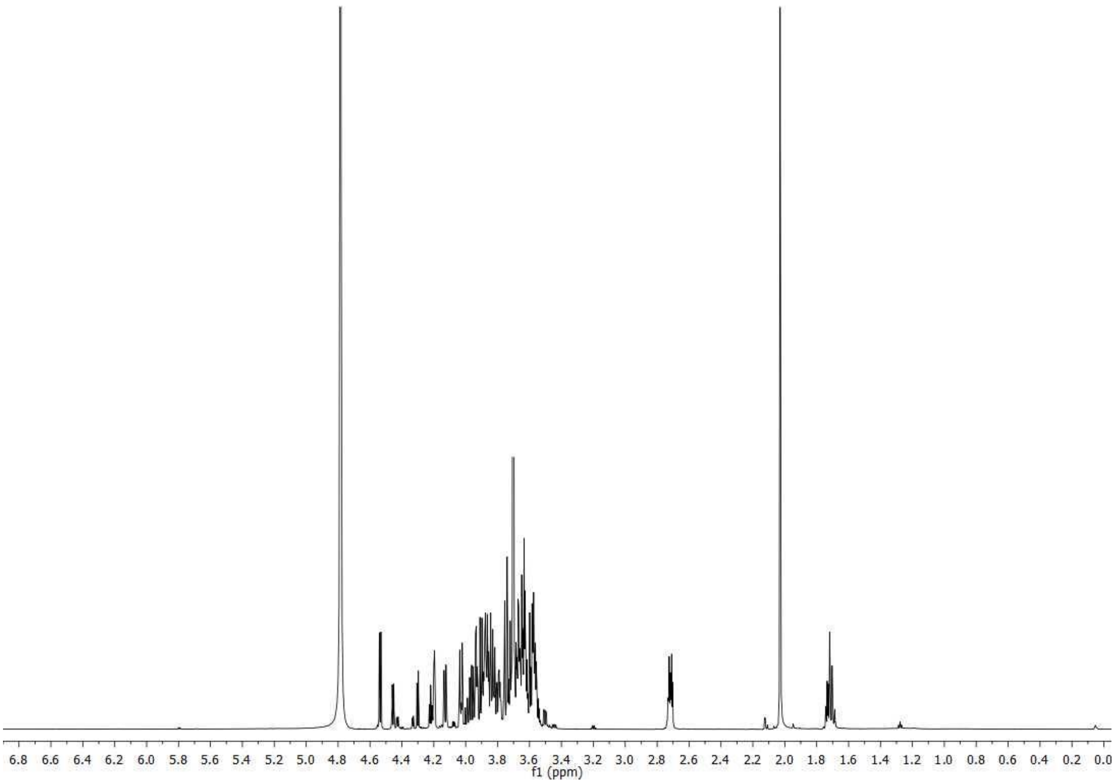
6.8 6.6 6.4 6.2 6.0 5.8 5.6 5.4 5.2 5.0 4.8 4.6 4.4 4.2 4.0 3.8 3.6 3.4 3.2 3.0 2.8 2.6 2.4 2.2 2.0 1.8 1.6 1.4 1.2 1.0 0.8 0.6 0.4 0.2 0.0
f1 (ppm)

B

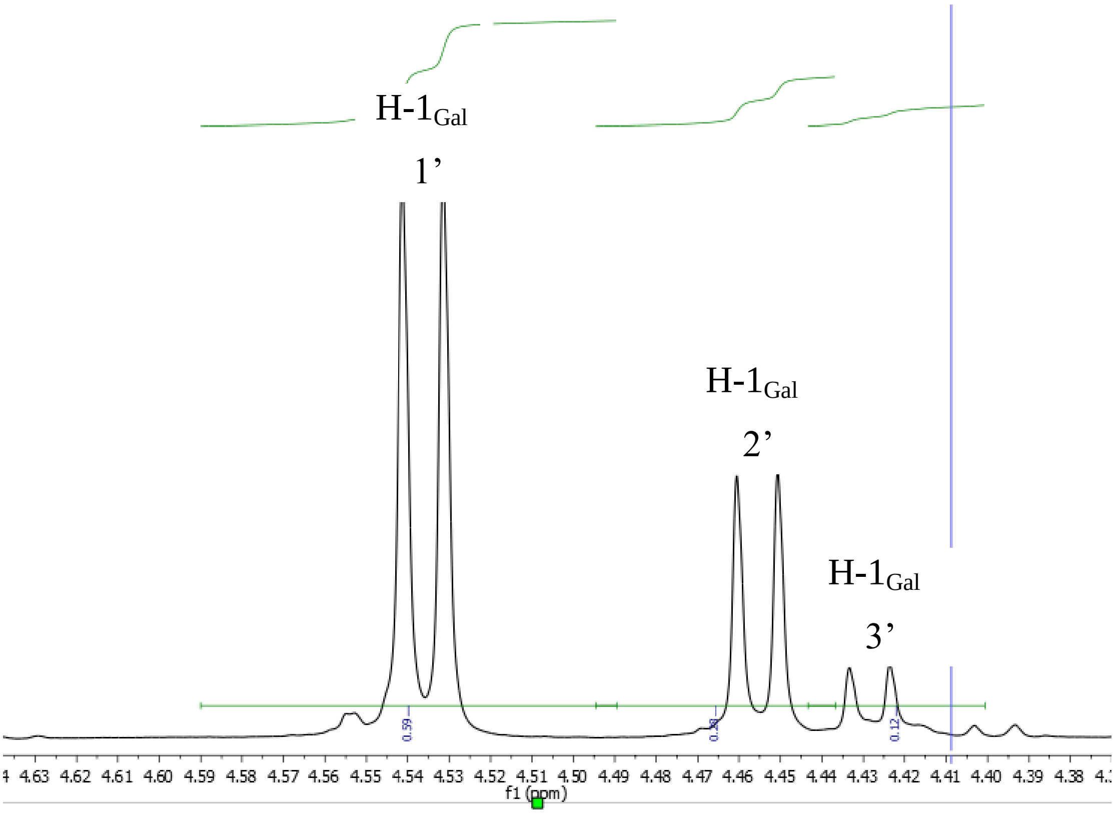
H-1Gal 1'
H-1Gal 2'
H-1Gal 3'
0.59
0.12
4.63 4.62 4.61 4.60 4.59 4.58 4.57 4.56 4.55 4.54 4.53 4.52 4.51 4.50 4.49 4.48 4.47 4.46 4.45 4.44 4.43 4.42 4.41 4.40 4.39 4.38
f1 (ppm)

C

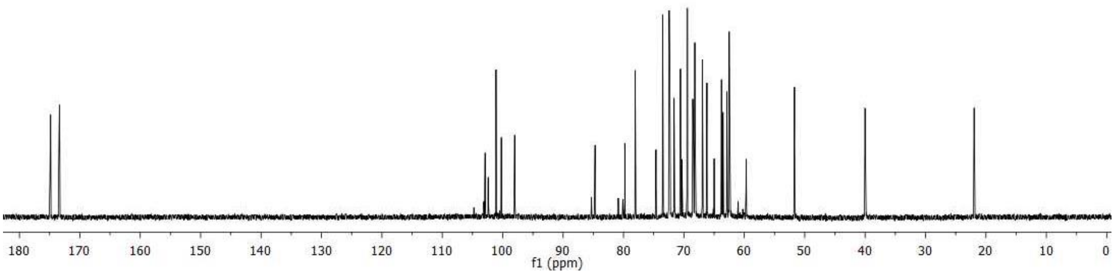

D

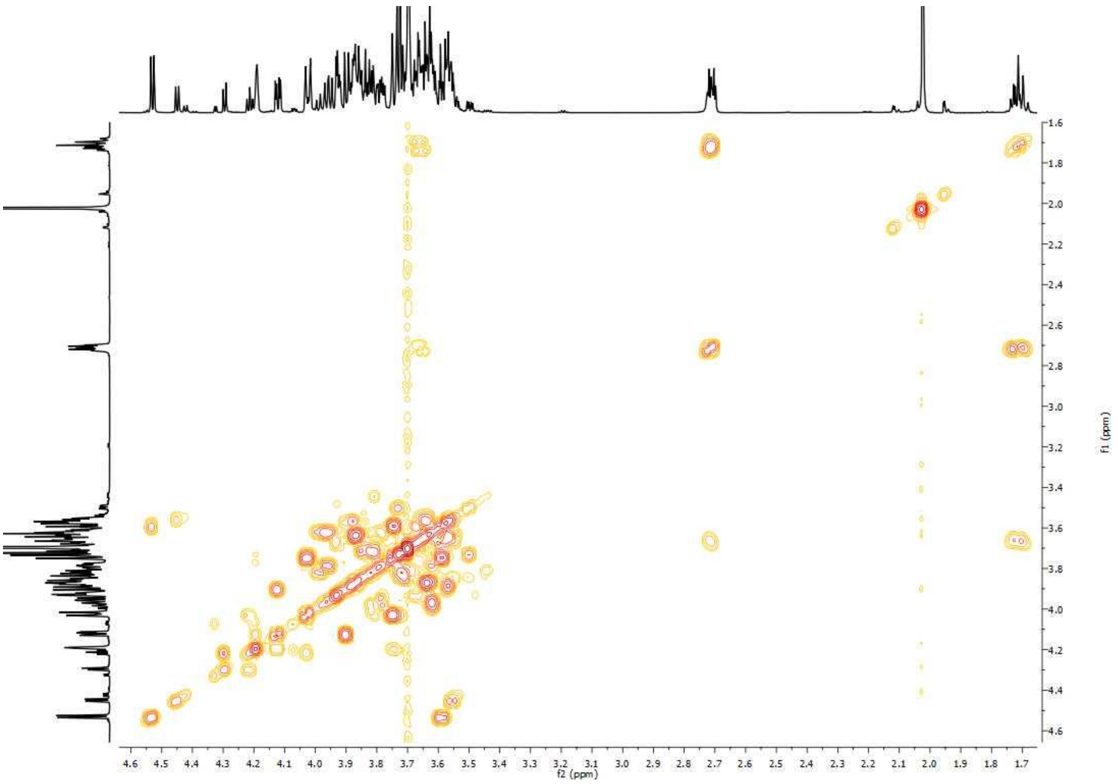

S3 Fig. ^{1}H, ^{13}C, and COSY NMR spectra of Neu5Ac-α2,6-lactulose. A, ^{1}H NMR spectra; B, zoomed ^{1}H NMR spectra; isomer 1’: Neu5Ac-α2,6-Gal-β1,4-β-D-fructopyranose; isomer 2’: Neu5Ac-α2,6-Gal-β1,4-β-D-fructopyranose; isomer 3’: Neu5Ac-α2,6-Gal-β1,4-α-D-fructopyranose, with ration 60%: 28%: 12%; C, ^{13}C NMR spectra; D, COSY NMR spectra.
