# Supplementary material for: Chemoenzymatic synthesis of sialylated lactuloses and their inhibitory effects on Staphylococcus aureus
Source: PLoS One. 2018 Jun 20;13(6):e0199334. doi: 10.1371/journal.pone.0199334 (PMC6010273; doi:10.1371/journal.pone.0199334)
Supplement: S1 Data — (PDF) [file pone.0199334.s004.pdf]

Supplementary Data

1) NMR data of Neu5Ac-α2,3-lactulose

^{1}H NMR (800 MHz, D_2O) δ 4.65 (d, *J* = 8.0 Hz, 0.7 H, H-1′_isomer 1), 4.55 (dd, *J* = 7.8 Hz, 0.2 H, H-1′_isomer 2), 4.53 (dd, *J* = 8.0 Hz, 0.1 H, H-1′_isomer 3), 4.32 (d, *J* = 4.0 Hz, 0.1 H, H-3_isomer 3), 4.29 (d, *J* = 7.2 Hz, 0.2 H, H-3_isomer 2), 4.27 (t, *J* = 7.2 Hz, 0.2 H, H-4_isomer 2), 4.23 (ddd, *J* = 3.2, 5.6, and 7.2 Hz, 0.1 H, H-5_isomer 3), 4.22 (dt, *J* = 1.6 and 3.2 Hz, 0.7 H, H-5_isomer 1), 4.15 (dd, *J* = 3.2 and 9.6 Hz, 0.7 H, H-4_isomer 1), 4.13 (dd, *J* = 3.2 and 9.6 Hz, 0.7 H, H-3′_isomer 1), 4.12 (dd, *J* = 3.2 and 9.6 Hz, 0.2 H, H-3′_isomer 2), 4.10 (dd, *J* = 3.2 and 9.6 Hz, 0.1 H, H-3′_isomer 3), 4.09 (dd, *J* = 4.0 and 7.2 Hz, 0.1 H, H-4_isomer 3), 4.04 (ddd, *J* = 3.2, 4.8, and 6.4 Hz, 0.2 H, H-5_isomer 2), 4.02 (dd, *J* = 1.6 and 13.6 Hz, 0.7 H, H-6a_isomer 1), 3.96 (bd, *J* = 4.0 Hz, 0.7 H, H-4′_isomer 1), 3.95 (bd, *J* = 4.0 Hz, 0.3 H, H-4′_isomer 2, H-4′_isomer 3), 3.92 (d, *J* = 9.6 Hz, 0.7 H, H-3_isomer 1), 3.90-3.81 (m, 3 H, H-5″_isomer 1/2/3, H-9a″_isomer 1/2/3, H-6a′_isomer 1/2/3), 3.79-3.64 (m, 6.3 H, H-8″_isomer 1/2/3, H-6b′_isomer 1/2/3, H-5′_isomer 1/2/3, H-6a_isomer 2/3, H-6b_isomer 1/2/3, H-1a_isomer 1/2/3, H-4″_isomer 1/2/3), 3.65-3.55 (m, 5 H, H-2′_isomer 1/2/3, H-1b_isomer 1/2/3, H-9b″_isomer 1/2/3, H-7″_isomer 1/2/3, H-6″_isomer 1/2/3), 2.78-2.75 (m, 1H, H-3$_{ea}$″_isomer 1/2/3), 2.03 (s, 3H), 1.80 (t, *J* = 12.0 Hz, 1H, H-3$_{ax}$″_isomer 1/2/3); ^{13}C NMR (200 MHz, D_2O) δ 174.90, 173.73, 102.83, 102.41, 102.26, 100.25, 99.71, 99.69, 97.96, 85.07, 83.99, 80.65, 80.50, 79.93, 77.18, 75.55, 75.47, 75.43, 75.05, 74.97, 74.95, 74.62, 72.75, 71.69, 71.66, 69.48, 69.11, 69.06, 68.25, 67.97, 67.42, 67.28, 67.25, 66.48, 65.97, 63.81, 62.85, 62.69, 62.54, 62.46, 62.42, 61.02, 60.91, 59.28, 51.58, 39.59, 39.53, 21.95.

2) NMR data of Neu5Ac-α2,6-lactulose:

^{1}H NMR (800 MHz, D_2O) δ 4.54 (d, *J* = 8.0 Hz, 0.6 H, H-1′_isomer 1’), 4.46 (dd, *J* = 7.8 Hz, 0.3 H, H-1′_isomer 2’), 4.43 (dd, *J* = 8.0 Hz, 0.1 H, H-1′_isomer 3’), 4.33 (d, *J* = 4.8 Hz, 0.1 H, H-3_isomer 3’), 4.30 (d, *J* = 7.2 Hz, 0.3 H, H-3_isomer 2’), 4.22 (t, *J* = 7.2 Hz, 0.3 H, H-4_isomer 2’), 4.20-4.19 (m, 0.7 H, H-5_isomer 2’/3’), 4.13 (dd, *J* = 3.2 and 10.4 Hz, 0.6 H, H-4_isomer 1’), 4.08 (dd, *J* = 4.8 and 7.2 Hz, 0.1 H, H-4_isomer 3’), 4.03 (ddd, *J* = 3.2, 4.8, and 6.4 Hz, 0.3 H, H-5_isomer 2’), 4.03 (dd, *J* = 1.6 and 12.8 Hz, 0.6 H, H-6a_isomer 1’), 4.00-3.95 (m, 1 H, H-6′_isomer 1’/2’/3’), 3.94 (d, *J* = 4.0 Hz, 0.6 H, H-4′_isomer 1’), 3.95 (d, *J* = 4.0 Hz, 0.4 H, H-4′_isomer 2’, H-4′_isomer 3’), 3.90 (d, *J* = 9.6 Hz, 0.6 H, H-3_isomer 1’), 3.89-3.78 (m, 4 H, H-5″_isomer 1’/2’/3’, H-9a″_isomer 1’/2’/3’, H-6a′_isomer 1’/2’/3’, H-3′_isomer 1’/2’/3’), 3.76-3.61 (m, 7.4 H, H-8″_isomer 1’/2’/3’, H-5′_isomer 1’/2’/3’, H-6a_isomer 2’/3’, H-6b_isomer 1’/2’/3’, H-1a_isomer 1’/2’/3’, H-4″_isomer 1’/2’/3’, H-6″_isomer 1’/2’/3’, H-9b″_isomer 1’/2’/3’), 3.60-3.49 (m, 3 H, H-2′_isomer 1’/2’/3’, H-1b_isomer 1’/2’/3’, H-7″_isomer 1’/2’/3’), 2.73 (dd, *J* = 4.8 and 12.8 Hz, 0.1 H, H-3$_{ea}$″_isomer 3’), 2.72 (dd, *J* = 4.8 and 12.8 Hz, 0.3 H, H-3$_{ea}$″_isomer 2’), 2.71 (dd, *J* = 4.8 and 12.8 Hz, 0.6 H, H-3$_{ea}$″_isomer 1’), 2.03 (s, 3H), 1.73 (t, *J* = 12.8 Hz, 0.3 H, H-3$_{ax}$″_isomer 2’), 1.72 (t, *J* = 12.8 Hz, 0.6 H, H-3$_{ax}$″_isomer 1’), 1.71 (t, *J* = 12.8 Hz, 0.1 H, H-3$_{ax}$″_isomer 3’); ^{13}C

NMR (200 MHz, D_2O) δ 174.94, 174.91, 174.84, 173.54, 173.37, 173.33, 173.32, 103.13, 102.91, 102.40, 101.10, 100.24, 100.22, 100.19, 98.01, 85.31, 84.70, 80.86, 80.11, 79.78, 78.07, 74.62, 73.53, 73.45, 72.52, 72.46, 72.44, 72.34, 72.25, 71.63, 71.59, 71.55, 70.57, 70.50, 70.45, 70.42, 70.33, 69.48, 68.53, 68.40, 68.23, 68.20, 68.19, 68.16, 68.14, 68.12, 68.10, 66.95, 66.19, 65.12, 64.99, 63.78, 63.65, 63.52, 62.90, 62.79, 62.53, 62.50, 62.47, 62.37, 62.31, 61.01, 59.69, 51.76, 51.72, 51.69, 40.01, 39.96, 39.93, 21.95, 21.93.
